# Quadratic descriptors and reduction methods in a two-layered model for compound inference

**DOI:** 10.3389/fgene.2024.1483490

**Published:** 2025-01-29

**Authors:** Jianshen Zhu, Naveed Ahmed Azam, Shengjuan Cao, Ryota Ido, Kazuya Haraguchi, Liang Zhao, Hiroshi Nagamochi, Tatsuya Akutsu

**Affiliations:** ^1^ Department of Applied Mathematics and Physics, Graduate School of Informatics, Kyoto University, Kyoto, Japan; ^2^ Department of Mathematics, Quaid-i-Azam University, Islamabad, Pakistan; ^3^ Graduate School of Advanced Integrated Studies in Human Survivability (Shishu-Kan), Kyoto University, Kyoto, Japan; ^4^ Bioinformatics Center, Institute for Chemical Research, Kyoto University, Uji, Japan

**Keywords:** machine learning, integer programming, chemo-informatics, materials informatics, QSAR/QSPR, molecular design

## Abstract

Compound inference models are crucial for discovering novel drugs in bioinformatics and chemo-informatics. These models rely heavily on useful descriptors of chemical compounds that effectively capture important information about the underlying compounds for constructing accurate prediction functions. In this article, we introduce quadratic descriptors, the products of two graph-theoretic descriptors, to enhance the learning performance of a novel two-layered compound inference model. A mixed-integer linear programming formulation is designed to approximate these quadratic descriptors for inferring desired compounds with the two-layered model. Furthermore, we introduce different methods to reduce descriptors, aiming to avoid computational complexity and overfitting issues during the learning process caused by the large number of quadratic descriptors. Experimental results show that for 32 chemical properties of monomers and 10 chemical properties of polymers, the prediction functions constructed by the proposed method achieved high test coefficients of determination. Furthermore, our method inferred chemical compounds in a time ranging from a few seconds to approximately 60 s. These results indicate a strong correlation between the properties of chemical graphs and their quadratic graph-theoretic descriptors.

## 1 Introduction

In recent years, extensive studies have been done on the design of novel molecules using various machine learning techniques (Lo et al., 2018; Tetko and Engkvist, 2020; Gartner III et al., 2024). Computational molecular design also has a long history in the field of chemo-informatics and has been studied under the names of *quantitative structure-activity relationship* (QSAR) (Cherkasov et al., 2014) and *inverse quantitative structure-activity relationship* (inverse QSAR) (Miyao et al., 2016; Ikebata et al., 2017; Rupakheti et al., 2015). This design problem has also become an important topic in both bioinformatics and machine learning.

The purpose of QSAR is to predict chemical activities from given chemical structures (Cherkasov et al., 2014). In most QSAR studies, a chemical structure is represented as a vector of real numbers called *features* or *descriptors*, and then a prediction function is applied to the vector, where a chemical structure is given as a *chemical graph*, which is defined by an undirected graph with an assignment of chemical elements to vertices and an assignment of bond multiplicities to edges. A prediction function is usually obtained from existing structure–activity relationship data. To this end, various regression-based methods have been utilized in traditional QSAR studies, whereas machine learning-based methods, including artificial neural network (ANN)-based methods, have recently been utilized (Ghasemi et al., 2018; Kim et al., 2021; Catacutan et al., 2024).

Conversely, the purpose of inverse QSAR is to predict chemical structures from given chemical activities (Miyao et al., 2016; Ikebata et al., 2017; Rupakheti et al., 2015; Miyao and Funatsu, 2024), where additional constraints may often be imposed to effectively restrict the possible structures. In traditional inverse QSAR, a feature vector is computed by applying some optimization or sampling method on the prediction function obtained by standard QSAR, and then chemical structures are reconstructed from the feature vector. However, the reconstruction itself is quite difficult due to the vast number of possible chemical graphs (Bohacek et al., 1996). In fact, inferring a chemical graph from a given feature vector, except for some simple cases, is an NP-hard problem (Akutsu et al., 2012). Due to this inherent difficulty, most existing methods employ heuristic methods for the reconstruction of chemical structures and thus do not guarantee optimal or exact solutions. On the other hand, one of the advantages of ANNs is that generative models, such as autoencoders and generative adversarial networks, are available. Furthermore, graph-structured data can be directly handled by using graph convolutional networks (Kipf and Welling, 2016). Therefore, it is reasonable to try to apply ANNs to inverse QSAR (Xiong et al., 2021). Various ANN models, including recurrent networks, autoencoders, generative networks, and invertible flow models, have been applied in this context (Segler et al., 2018; Yang et al., 2017; Gómez-Bombarelli et al., 2018; Kusner et al., 2017; De Cao and Kipf, 2018; Madhawa et al., 2019; Shi et al., 2020). However, the optimality or exactness of the solutions provided by these methods is not yet guaranteed.

A novel two-phase framework based on mixed-integer linear programming (MILP) and machine learning methods has been developed to infer chemical graphs (Shi et al., 2021; Zhu et al., 2022b; Ido et al., 2021; Azam et al., 2021a). The first phase constructs a prediction function η for a chemical property, and the second phase infers a chemical graph with a target value of the property based on the function η . For a chemical property π , we define $C_{\pi }$ as a data set of chemical graphs such that the observed value a(C) of property π for every chemical graph $C\in C_{\pi }$ is available.

In the first phase, we introduce a feature function $f:G\to R^{K}$ for a positive integer K , where the descriptors of a chemical graph are defined based on local graph structures by using a *two-layered model* (Shi et al., 2021) so that the inverse of f can be modeled by MILP in the second phase. The prediction function aims to produce an output y=η(x)∈R based on the feature vector $x=f\left(C\right)\in R^{K}$ for each $C\in C_{\pi }$ . This output serves as a predicted value for the real value a(C) .

**FIGURE 1:**
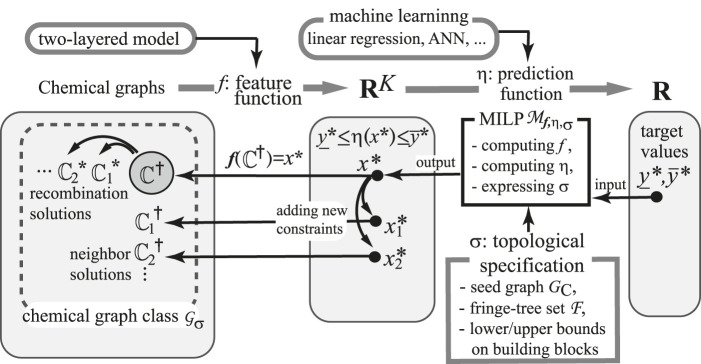
An illustration of inferring desired chemical graphs $C\in G_{\sigma }$ with $\underline{y^{*}}\leq \eta \left(f\left(C\right)\right)\leq \bar{y^{*}}$ .

The task of the second phase is to infer desired chemical graphs. This phase consists of three steps. For a given set of rules called *topological specification* σ and a range $\left[{\underline{y}}^{*},{\bar{y}}^{*}\right]$ of the target property value, the aim of the first step is to infer chemical graphs $C^{*}$ that satisfy the rules σ and ${\underline{y}}^{*}\leq \eta \left(f\left(C^{*}\right)\right)\leq {\bar{y}}^{*}$ (see Figure 1 for an illustration). For this, we formulate an MILP $M_{f,\eta ,\sigma }$ that represents (i) the computation process of x≔f(C) from a chemical graph C in the feature function f ; (ii) that of y≔η(x) from a vector $x\in R^{K}$ in the prediction function η ; and (iii) the constraint $C\in G_{\sigma }$ , where $G_{\sigma }$ denotes the set of all chemical graphs that satisfy the rules in σ . Given an interval with ${\underline{y}}^{*},{\bar{y}}^{*}\in R$ , we solve the MILP $M_{f,\eta ,\sigma }$ to find a feature vector $x^{*}\in R^{K}$ and a chemical graph $C^{†}\in G_{\sigma }$ such that $f\left(C^{†}\right)=x^{*}$ and ${\underline{y}}^{*}\leq \eta \left(x^{*}\right)\leq {\bar{y}}^{*}$ (if the MILP instance is infeasible, this suggests that $G_{\sigma }$ does not contain such a desired chemical graph). See Zhu et al. (2022a) for a full description of the framework.

In the second and third steps of the process, we employ different techniques to generate additional desired chemical graphs based on the initial solution $C^{†}$ . These techniques include a dynamic programming algorithm (Azam et al., 2021b) and a neighbor search method (Azam et al., 2021a). The dynamic programming algorithm operates by decomposing $C^{†}$ into trees and generating their isomers. These isomers are then combined to produce a set of isomers $C^{*}$ that belong to the desired chemical graph space $G_{\sigma }$ . These isomers are referred to as the *recombination solutions* of $C^{†}$ . The idea of the neighbor search method is to generate new desired chemical graphs $C_{i}^{†}\in G_{\sigma }$ that have a slightly different feature vector than that of $C^{†}$ . For this purpose, we solve an augmented MILP obtained by $M_{f,\eta ,\sigma }$ with an additional set of linear constraints. The resulting graphs obtained through this process are referred to as the *neighbor solutions* of $C^{†}$ .

Contribution: The feature vector x=f(C) of a chemical graph C in this framework consists of the descriptors x(i),1≤i≤K that extract the local information of the interior and exterior parts of the graph obtained from the two-layered model. These simple descriptors play a crucial role in deriving compact MILP formulations for the inference of a desired chemical graph. However, for certain chemical properties, the prediction functions constructed based on the feature function f could not achieve evaluation scores in the acceptable range. To improve the evaluation score, we introduce new *quadratic descriptors* x(i)x(j) (or x(i)(1−x(j)) . This drastically increases the number of descriptors, which would take extra running time for learning or lead to overfitting of the data set. Moreover, computed quadratic descriptors cannot be directly formulated as a set of linear constraints in the original MILP. For this, we introduce a method of reducing a set of descriptors into a smaller set that delivers a prediction function with a higher performance. We also design an MILP formulation to represent a quadratic term x(i)x(j) . Based on the same MILP $M_{f,\eta ,\sigma }$ formulation proposed by Zhu et al. (2022b), we implemented the framework to treat the feature function with quadratic descriptors. From the results of our computational experiments on more than 40 chemical properties, we observe that our new method of utilizing quadratic descriptors has improved the performance of a prediction function for many chemical properties.

## 2 Quadratic descriptors and their approximation in an MILP

In the framework with the two-layered model (Shi et al., 2021), the feature vector consists of graph-theoretic descriptors that are mainly the frequencies of atoms, bonds, and edge configurations in the interior and exterior of the chemical compounds. See Zhu et al. (2022a) for the details of the interior and exterior of chemical compounds and all the descriptors $x\left(1\right),x\left(2\right),\ldots ,x\left(K_{1}\right)$ , which are called *linear descriptors*, respectively. There are some chemical properties for which the performance of a prediction function constructed with this feature vector remains rather low. To improve the learning performance in the two-layered model, we introduce quadratic terms x(i)x(j) (or x(i)(1−x(j)) ) and $1\leq i\leq j\leq K_{1}$ as a new descriptor, where we assume that each x(i) is normalized between 0 and 1 by using the min-max normalization. Note that computing quadratic descriptors cannot be directly formulated as a set of linear constraints in the original MILP used in the two-layered model. Therefore, this section introduces an MILP formulation that approximates the product of two descriptors in a MILP.

Given two real values x and y with 0≤x≤1 and 0≤y≤1 , the process of computing the product z=xy can be approximately formulated as the following MILP. First, regard $\left(2^{p+1}-1\right)x$ as an integer with a binary expression of p+1 bits, where $x^{\left(j\right)}\in \left[0,1\right]$ denotes the value of the j -th bit. Then, compute $y\cdot x^{\left(j\right)}$ , which becomes the j -th bit $z^{\left(j\right)}$ of $\left(2^{p+1}-1\right)z$ .

Constants:

- x,y : reals with 0≤x,y≤1 ;
- p : a positive integer;

variables:

- z , $z^{\left(j\right)},j\in \left[0,p\right]$ : reals with $0\leq z,z^{\left(j\right)}\leq 1$ ;
- $x^{\left(j\right)}\in \left[0,1\right],j\in \left[0,p\right]$ : binary variables;

constraints:

$$\begin{array}{ll} \underset{j\in \left[0,p\right]}{\sum }2^{j}x^{\left(j\right)}-1\leq \left(2^{p+1}-1\right)x\leq \underset{j\in \left[0,p\right]}{\sum }2^{j}x^{\left(j\right)}, & \\ z^{\left(j\right)}\leq x^{\left(j\right)}, & \;j\in \left[0,p\right],\\ y-\left(1-x^{\left(j\right)}\right)\leq z^{\left(j\right)}\leq y+\left(1-x^{\left(j\right)}\right), & \;j\in \left[0,p\right],\\ z=\frac{1}{2^{p+1}-1}\underset{j\in \left[0,p\right]}{\sum }2^{j}z^{\left(j\right)}. \end{array}$$

Note that the necessary number of integer variables for computing xy for one pair of x and y is p . In this article, we set p≔6 in our computational experiment. The relative error by p=6 in the above method is at most $\frac{1}{2^{p+1}-1}=1/127$ , which is approximately 0.8% .

## 3 Methods for reducing descriptors

The number of descriptors in the two-layered model increases drastically due to the quadratic descriptors. More precisely, if $D_{\pi }^{\left(1\right)}≔\left\{x\left(k\right)|k\in \left[1,K_{1}\right]\right\}$ denotes the set of linear descriptors, and $D_{\pi }^{\left(2\right)}≔\left\{x\left(i\right)x\left(j\right)|1\leq i\leq j\leq K_{1}\right\}\cup \left\{x\left(i\right)\left(1-x\left(j\right)\right)|1\leq i,j\leq K_{1}\right\}$ denotes the set of quadratic descriptors constructed over a data set for a property π , then the total number of descriptors are $K_{1}+\left(3{\left(K_{1}\right)}^{2}+K_{1}\right)/2$ . This large number of descriptors would increase the running time to construct prediction functions or lead to overfitting of the data set. To address these issues, we propose methods for reducing descriptors in this section.

Let C be a given set of chemical compounds, D be a descriptor set obtained using the two-layered model, and $K^{*}\in \left[1,|D|\right]$ be the size of a set of selected descriptors.

For given C , D and a real number λ>0 , we denote by Des-set-LLR (C,D,λ) the subset S of D such that w(d)=0 for d∈S and the hyperplane (w,b) constructed by the LLR procedure LLR (C,D,λ) .

### 3.1 A method based on lasso linear regression

Because the lasso linear regression finds some number of descriptors d∈D with w(d)=0 in the output hyperplane (w,b) , we can reduce a given set of descriptors by applying the lasso linear regression repeatedly. Choose parameters $c_{\max}$ and $d_{\max}$ so that LLR (C,D,λ) can be executed in a reasonable running time when $|C|\leq c_{\max}$ and $|D|\leq d_{\max}$ . Let $\tilde{K}\in \left[1,|D|\right]$ be an integer for the number of descriptors that we choose from a given set D of descriptors.

LLR-Reduce (C,D) :

Input: A data set C and a set D of descriptors;
Output: A subset $\tilde{D}\subseteq D$ with $|\tilde{D}|=\tilde{K}$ .
Initialize $D^{\prime }≔D$ ;
**while** $|D^{\prime }|>\tilde{K}$ **do**
 Partition $D^{\prime }$ randomly into disjoint subsets $D_{1},D_{2},\ldots ,D_{p}$ such that
  
$|D_{i}|\leq d_{\max}$ for each i ;
 **for** each i=1,2,…,p **do**
  Choose a subset $C_{i}$ with $|C_{i}|=\min\left\{c_{\max},|C|\right\}$ of C randomly;
  
$D_{i}^{\prime }≔$ Des-set-LLR $\left(C_{i},D_{i},\lambda \right)$ for some λ>0

 **end for**;

$D^{\prime }≔D_{1}^{\prime }\cup D_{2}^{\prime }\cup \cdots \cup D_{p}^{\prime }$

**end while**;
Output $\tilde{D}≔D^{\prime }$ after adding to $D^{\prime }$ extra $\tilde{K}-|D^{\prime }|$ descriptors from the previous
set $D^{\prime }$ when $|D^{\prime }|<\tilde{K}$ by using the K-best method.

In this article, we set $c_{\max}≔200$ , $d_{\max}≔200$ , $\tilde{K}≔5000$ and w(d)≔0 if $|w\left(d\right)|\leq 10^{-6}$ in our computational experiment.

### 3.2 A method based on the backward stepwise procedure

A backward stepwise procedure (Draper and Smith, 1998) reduces the number of descriptors one by one, choosing the one removal that maximizes the learning performance and outputs a subset with the maximum learning performance among all subsets during the reduction iteration.

For a subset S⊆D and a positive integer p , let $R_{\text{CV,MLR}}^{2}\left(C,S,p\right)$ denote the median of the coefficient of determination $R^{2}$ test score of a prediction function $\eta_{w,b}$ by MLR (C,S) constructed during p times 5-fold cross-validations. We define a performance evaluation function $g_{p}:2^{D}\to R$ for an integer p≥1 such that $g_{p}\left(S\right)=R_{\text{CV,MLR}}^{2}\left(C,S,p\right)$ . The backward stepwise procedure with this function $g_{p}$ is described as follows.

BS-Reduce (C,D,p) :

Input: A data set C , a set D of descriptors, an integer p≥1 , and a performance evaluation function $g_{p}:2^{D}\to R$ defined above;
Output: A subset $D^{*}\subseteq D$ .
Compute $\ell_{\text{best}}≔g_{p}\left(D\right)$ ; Initialize $D_{\text{best}}≔D^{\prime }≔D$ ;
**while** $D^{\prime }\neq \emptyset$ **do**
 Compute $\ell \left(d\right)≔g_{p}\left(D^{\prime }\backslash \left\{d\right\}\right)$ for each descriptor $d\in D^{\prime }$ ;
 Set $d^{*}\in D^{\prime }$ to be a descriptor that maximizes ℓ(d) over all $d\in D^{\prime }$ ;
 Update: $D^{\prime }≔D^{\prime }\backslash \left\{d^{*}\right\}$ ;
 **if** $\ell \left(d^{*}\right)>\ell_{\text{best}}$ **then** update $D_{\text{best}}≔D^{\prime }$ and $\ell_{\text{best}}≔\ell \left(d^{*}\right)$ **end if**
**end while**;
Output $D^{*}≔D_{\text{best}}$ .

Based on the lasso linear regression and the backward stepwise procedure, we design the following method for choosing a subset $D^{*}$ of a given set D of descriptors. We are given a set A of 17 real numbers and a set B(a) of 16 real numbers close to each number a∈A . The method first chooses a best parameter $\lambda_{\text{best}}\in A$ to construct a prediction function by LLR and then chooses a subset $D_{i}\subseteq D$ for each $\lambda_{i}\in B\left(\lambda_{\text{best}}\right)$ by the backward stepwise procedure. The procedure takes $O\left(|D{|}^{2}\right)$ iterations, which may take a large amount of running time. We introduce an upper bound $s_{\max}$ on the size of an input descriptor set D for the backward stepwise procedure. Let $p_{1}$ , $p_{2}$ , and $p_{3}$ be integer parameters that control the number of executions of cross-validations to evaluate the learning performance in the method.

Select-Des-set (C,D) :

Input: A data set C , a set D of descriptors, a set A of different values of λ , and a set B(λ) of real numbers close to each λ∈A ;
Output: A subset $D^{*}$ of D .
**for** each λ∈A **do**
 Compute $D_{\lambda }≔$ Des-set-LLR (C,D,λ) and $\ell_{\lambda }≔R_{\text{CV,MLR}}^{2}\left(C,D_{\lambda },p_{1}\right)$

**end for**;
Set $\lambda_{\text{best}}$ to be a λ∈A that maximizes $\ell_{\lambda }$ ; Denote $B\left(\lambda_{\text{best}}\right)$ by $\left\{\lambda_{1},\lambda_{2},\ldots ,\lambda_{16}\right\}$ ;
**for** each i∈[1,16] **do**
 Compute $D_{i}≔$ Des-set-LLR $\left(C,D,\lambda_{i}\right)$ and let $\left(w,b\right),w\in R^{\left|D\right|},b\in R$ be the hyperplane obtained by this LLR;
 **if** $|D_{i}|\leq s_{\max}$ **then**
   
$D_{i}^{\prime }≔D_{i}$

 **else**
  Let $D_{i}^{\prime }$ consist of $s_{\max}$ descriptors $d\in D_{i}$ that have the $s_{\max}$ largest absolute
  values |w(d)| in the weight sets $\left\{w\left(d\right)|d\in D_{i}\right\}$ of the hyperplane (w,b)

 **end if**;
  
$D_{i}^{†}≔$ BS-Reduce $\left(C,D_{i}^{\prime },p_{2}\right)$ ; $\ell_{i}≔R_{\text{CV,MLR}}^{2}\left(C,D_{i}^{†},p_{3}\right)$

**end for**;
Output $D^{*}$ to be a set $D_{i}^{†}$ that maximizes $\ell_{i},i\in \left[1,16\right]$ .

In the computational experiment in this article, we set A= { $0,10^{-6},10^{-5},10^{-4},10^{-3},0.01,0.05$ , 0.1,0.5,0.75,1,2,5,10,25,50,100 }, |B(λ)|=16 for each λ∈A , $p_{1}≔p_{2}≔p_{3}≔5$ , and $s_{\max}≔150+10^{4}/\left(|C|+200\right)$ .

## 4 Results and discussion

With our new method of choosing descriptors and formulating an MILP to treat quadratic descriptors in the two-layered model, we implemented the framework for inferring chemical graphs and conducted experiments to evaluate the computational efficiency.

### 4.1 Chemical properties

In the first phase, we used the following 32 chemical properties of monomers and ten chemical properties of polymers.

For monomers, we used the following data sets: biological half-life (BHL), boiling point (Bp), critical temperature (Ct), critical pressure (Cp), dissociation constants (Dc), flash point in closed cup (Fp), heat of combustion (Hc), heat of vaporization (Hv), octanol/water partition coefficient (Kow), melting point (Mp), optical rotation (OptR), refractive index of trees (RfIdT), vapor density (Vd) and vapor pressure (Vp) provided by HSDB from PubChem (2022); electron density on the most positive atom (EDPA) and Kovats retention index (Kov) provided by Jalali-Heravi and Fatemi (2001); entropy (ET) provided by Duchowicz et al. (2002); heat of atomization (Ha) and heat of formation (Hf) provided by Roy and Saha (2003); surface tension (SfT) by Goussard et al. (2017); viscosity (Vis) provided by Goussard et al. (2020); isobaric heat capacities liquid (LhcL) and isobaric heat capacities solid (LhcS) provided by Naef (2019); lipophilicity (Lp) provided by Xiao (2017); flammable limits lower of organics (FlmLO) provided by Yuan et al. (2019); molar refraction at 20° (Mr) provided by Ponce (2003); and solubility (Sl) provided by ESOL (MoleculeNet, 2022), and energy of highest occupied molecular orbital (Homo), energy of lowest unoccupied molecular orbital (Lumo), the energy difference between Homo and Lumo (Gap), isotropic polarizability (Alpha), heat capacity at 298.15 K (Cv), internal energy at 0 K (U0), and electric dipole moment (mu) provided by ESOL (MoleculeNet, 2022), where the properties from Homo to mu are based on a common data set QM9.

The data set QM9 contains more than 130,000 compounds. In our experiment, we used a set of 1,000 compounds randomly selected from the data set. For the Hv property, we removed the chemical compound with the compound identifier (CID) =7947 as an extremal outlier from the original data set.

For polymers, we used the following data provided by Bicerano (2002): experimental amorphous density (AmD), characteristic ratio (ChaR), dielectric constant (DieC), dissipation factor (DisF), heat capacity in liquid (HcL), heat capacity in solid (HcS), mol volume (MlV), permittivity (Prm), refractive index of polymers (RfIdP), and glass transition (Tg), where we excluded from our test data set every polymer whose chemical formula could not be found by its name in the book (Bicerano, 2002). A summary of these properties is given in Tables 1, 2. We remark that the previous learning experiments for π∈ { ChaR, RfIdP} based on the two-layered model proposed by Azam et al. (2021a) and Zhu et al. (2022c) excluded some number of polymers as outliers. In our experiments, we do not exclude any polymer from the original data set as outliers for these properties.

**TABLE 1:** Results of setting data sets for monomers.

π	Λ	$\left|C_{\pi }\right|$	$\underline{n},\;\bar{n}$	$\underline{a},\;\bar{a}$	|Γ|	|F|	$K_{1}$
Biological half life (BHL) (PubChem, 2022)	$\Lambda_{7}$	514	5, 36	0.03, 732.99	26	101	166
Boiling point (Bp) (PubChem, 2022)	$\Lambda_{2}$	370	4, 67	−11.7, 470.0	22	130	184
Boiling point (Bp) (PubChem, 2022)	$\Lambda_{7}$	444	4, 67	−11.7, 470.0	26	163	230
Critical pressure (Cp) (PubChem, 2022)	$\Lambda_{5}$	131	4, 63	$4.7\;\times \;10^{-6}$ , 5.52	8	79	119
Critical temperature (Ct) (PubChem, 2022)	$\Lambda_{2}$	125	4, 63	56.1, 3607.5	8	76	113
Critical temperature (Ct) (PubChem, 2022)	$\Lambda_{5}$	132	4, 63	56.1, 3607.5	8	81	121
Dissociation constants (Dc) (PubChem, 2022)	$\Lambda_{2}$	141	5, 44	0.5, 17.11	20	62	111
Dissociation constants (Dc) (PubChem, 2022)	$\Lambda_{7}$	161	5, 44	0.5, 17.11	25	69	130
Entropy (ET) (Duchowicz et al., 2002)	$\Lambda_{7}$	17	5, 12	64.34, 96.21	5	17	53
Flash point in closed cup (Fp) (PubChem, 2022)	$\Lambda_{2}$	368	4, 67	−82.99, 300.0	20	131	183
Flash point in closed cup (Fp) (PubChem, 2022)	$\Lambda_{7}$	424	4, 67	−82.99, 300.0	25	161	229
Flammable limits lower of organics (FlmLO) (Yuan et al., 2019)	$\Lambda_{16}$	1046	1, 49	0.185, 4.3	34	282	376
Heat of vaporization (Hv) (PubChem, 2022)	$\Lambda_{2}$	94	4, 16	19.12, 210.3	12	63	105
Kovats retention index (Kov) (Jalali-Heravi and Fatemi, 2001)	$\Lambda_{1}$	52	11, 16	1422.0, 1919.0	9	33	64
Octanol/water partition coefficient (Kow) (PubChem, 2022)	$\Lambda_{2}$	684	4, 58	−7.5, 15.6	25	166	223
Octanol/water partition coefficient (Kow) (PubChem, 2022)	$\Lambda_{8}$	899	4, 69	−7.5, 15.6	37	219	303
Lipophilicity (Lp) (Xiao, 2017)	$\Lambda_{2}$	615	6, 60	−3.62, 6.84	32	116	186
Lipophilicity (Lp) (Xiao, 2017)	$\Lambda_{8}$	936	6, 74	−3.62, 6.84	44	136	231
Melting point (Mp) (PubChem, 2022)	$\Lambda_{2}$	467	4, 122	−185.33, 300.0	23	142	197
Melting point (Mp) (PubChem, 2022)	$\Lambda_{8}$	577	4, 122	−185.33, 300.0	32	176	255
Optical rotation (OptR) (PubChem, 2022)	$\Lambda_{2}$	147	5, 44	−117.0, 165.0	21	55	107
Optical rotation (OptR) (PubChem, 2022)	$\Lambda_{4}$	157	5, 69	−117.0, 165.0	25	62	123
Refractive index of trees (RfIdT) (PubChem, 2022)	$\Lambda_{10}$	191	4, 26	0.919, 1.613	17	115	168
Solubility (Sl) (MoleculeNet, 2022)	$\Lambda_{2}$	673	4, 55	−9.332, 1.11	27	154	217
Solubility (Sl) (MoleculeNet, 2022)	$\Lambda_{8}$	915	4, 55	−11.6, 1.11	42	207	300
Surface tension (SfT) (Goussard et al., 2017)	$\Lambda_{3}$	247	5, 33	12.3, 45.1	11	91	128
Viscosity (Vis) (Goussard et al., 2017)	$\Lambda_{3}$	282	5, 36	−0.64, 1.63	12	88	126
Energy of highest-occupied molecular orbital (Homo) (MoleculeNet, 2022)	$\Lambda_{9}$	977	6, 9	−0.3335, −0.1583	59	190	297
Energy of lowest-unoccupied molecular orbital (Lumo) (MoleculeNet, 2022)	$\Lambda_{9}$	977	6, 9	−0.1144, 0.1026	59	190	297
The energy difference between Homo and Lumo (Gap) (MoleculeNet, 2022)	$\Lambda_{9}$	977	6, 9	0.1324, 0.4117	59	190	297
Isotropic polarizability (Alpha) (MoleculeNet, 2022)	$\Lambda_{9}$	977	6, 9	50.9, 99.6	59	190	297
Heat capacity at 298.15 K (Cv) (MoleculeNet, 2022)	$\Lambda_{9}$	977	6, 9	19.2, 44.0	59	190	297
Electric dipole moment (mu) (MoleculeNet, 2022)	$\Lambda_{9}$	977	6, 9	0.04, 6.897	59	190	297

**TABLE 2:** Results of setting data sets for polymers.

π	Λ	$\left|C_{\pi }\right|$	$\underline{n},\;\bar{n}$	$\underline{a},\;\bar{a}$	|Γ|	|F|	$K_{1}$
Experimental amorphous density (AmD) (Bicerano, 2002)	$\Lambda_{2}$	86	4, 45	0.838, 1.34	16	25	83
Experimental amorphous density (AmD) (Bicerano, 2002)	$\Lambda_{13}$	93	4, 45	0.838, 1.45	18	30	94
Characteristic ratio (ChaR) (Bicerano, 2002)	$\Lambda_{2}$	30	4, 18	3.7, 15.9	15	17	68
Characteristic ratio (ChaR) (Bicerano, 2002)	$\Lambda_{12}$	32	4, 18	3.7, 15.9	15	18	71
Characteristic ratio (ChaR) (Bicerano, 2002)	$\Lambda_{6}$	35	4, 18	3.7, 15.9	18	21	83
Dielectric constant (DieC) (Bicerano, 2002)	$\Lambda_{12}$	36	4, 22	2.13, 3.4	11	18	67
Dissipation factor (DisF) (Bicerano, 2002)	$\Lambda_{13}$	132	4, 45	$7\;\times \;10^{-5}$ , 0.07	15	18	78
Permittivity (Prm) (Bicerano, 2002)	$\Lambda_{2}$	112	4, 45	2.23, 4.91	14	15	69
Permittivity (Prm) (Bicerano, 2002)	$\Lambda_{13}$	132	4, 45	2.23, 4.91	15	18	78
Refractive index of polymers (RfIdP) (Bicerano, 2002)	$\Lambda_{11}$	92	4, 29	0.4899, 1.683	15	35	96
Refractive index of polymers (RfIdP) (Bicerano, 2002)	$\Lambda_{14}$	125	4, 29	0.4899, 1.683	19	50	124
Refractive index of polymers (RfIdP) (Bicerano, 2002)	$\Lambda_{15}$	135	4, 29	0.4899, 1.71	23	56	144
Glass transition (Tg) (Bicerano, 2002)	$\Lambda_{2}$	204	4, 58	171, 673	19	36	101
Glass transition (Tg) (Bicerano, 2002)	$\Lambda_{7}$	232	4, 58	171, 673	21	43	118

### 4.2 Experimental setup and setting data set

We executed the experiments on a PC with a Core i7-9700 (3.0 GHz; 4.7 GHz at the maximum processor and 16 GB RAM DDR4 memory. Prediction functions were constructed using scikit-learn version 1.0.2 with Python 3.8.12, and MLPRegressor and rectified linear unit (ReLU) activation function were used for learning based on ANN. The prediction functions constructed by different machine learning models were evaluated using the coefficient of determination $R^{2}$ . We performed 10 rounds of 5-fold cross-validation, calculated the training and testing $R^{2}$ scores for each cross-validation, and recorded the median of the testing $R^{2}$ scores across all 50 cross-validations.

For each property π , we first selected a set Λ of chemical elements and then collected a data set $C_{\pi }$ on chemical graphs over the set Λ of chemical elements. To construct the data set $C_{\pi }$ , we eliminated chemical compounds that did not satisfy one of the following: the graph is connected, the number of carbon atoms is at least four, and the number of non-hydrogen neighbors of each atom is at most four.

We set a branch-parameter ρ to be 2, introduce linear descriptors defined by the two-layered graph in the chemical model without suppressing hydrogen, and use the set $D_{\pi }^{\left(1\right)}\cup D_{\pi }^{\left(2\right)}$ of linear and quadratic descriptors (see Zhu et al. (2022a) for the details). We normalize the range of each linear descriptor and the range $\left\{t\in R|\underline{a}\leq t\leq \bar{a}\right\}$ of property values $a\left(C\right),C\in C_{\pi }$ by using the min–max normalization.

Among the above properties, we found that the median of the test coefficient of determination $R^{2}$ of the prediction function constructed by LLR (Zhu et al., 2022b) or ALR (Zhu et al., 2022c) exceeds 0.98 for the following nine properties of monomers (resp., three properties of polymers): EDPA, Hc, Ha, Hf, LhcL, LhcS, Mr, Vd, and U0 (resp., HcL, HcS, and MlV). We excluded the above properties from further analysis because they already achieved excellent predictive performance, and further improvement would not provide additional insights. The remaining 23 chemical properties of monomers and seven chemical properties of polymers were used in the following experiments.

Tables 1, 2 show the size and range of data sets that we prepared for each chemical property to construct a prediction function, where we denote the following:

- π : the name of a chemical property used in the experiment.
- Λ : a set of selected elements used in the data set $C_{\pi }$ ; Λ is one of the following 19 sets:
       
$\Lambda_{1}=$ {H,C,O}; $\Lambda_{2}=$ {H,C,O, N}; $\Lambda_{3}=$ {H,C,O, Si_(4)_ }; $\Lambda_{4}=$ {H,C,O, N,S_(2)_,F}; $\Lambda_{5}=$ {H,C,O, N, Cl, Pb}; $\Lambda_{6}=$ {H,C,O, N,Si_(4)_,Cl,Br}; $\Lambda_{7}=$ {H,C,O, N,S_(2)_,S_(6)_,Cl}; $\Lambda_{8}=$ {H,C,O, N,S_(2)_,S_(4)_,S_(6)_,Cl}; $\Lambda_{9}=$ {H, C_(2)_,C_(3)_,C_(4)_,C_(5)_,O, N_(1)_, N_(2)_, N_(3)_, F}; $\Lambda_{10}=$ {H,C,O, N,P_(2)_,P_(5)_,Cl}; $\Lambda_{11}=$ {H, C, O_(1)_, O_(2)_, N}; $\Lambda_{12}=$ {H, C, O, N, Cl}; $\Lambda_{13}=$ {H, C, O, N, Cl, S_(2)_ }; $\Lambda_{14}=$ {H, C, O_(1)_, O_(2)_, N, Cl, Si_(4)_, F}; $\Lambda_{15}=$ {H,C,O_(1)_, O_(2)_, N, Si_(4)_,Cl,F
      S_(2)_, S_(6)_,Br}; $\Lambda_{16}=$ {H, C, O_(2)_, N, Cl, P_(3)_, P_(5)_, S_(2)_, S_(4)_, S_(6)_, Si_(4)_, Br, I}, where a_(*i*)_ for a chemical element a, and an integer i≥1 means a chemical element a with valence i .
- $\left|C_{\pi }\right|$ : the size of data set $C_{\pi }$ over Λ for the property π .
- $\underline{n},\;\bar{n}$ : the minimum and maximum values of the number n(C) of non-hydrogen atoms in the compounds C in $C_{\pi }$ .
- $\underline{a},\;\bar{a}$ : the minimum and maximum values of a(C) for π over the compounds C in $C_{\pi }$ .
- |Γ| : the number of different edge-configurations of interior edges over the compounds in $C_{\pi }$ .
- |F| : the number of non-isomorphic chemical rooted trees in the set of all 2-fringe-trees in the compounds in $C_{\pi }$ .
- $K_{1}$ : the size $\left|D_{\pi }^{\left(1\right)}\right|$ of a set $D_{\pi }^{\left(1\right)}$ of linear descriptors, where $|D_{\pi }^{\left(2\right)}|=\left(3{\left(K_{1}\right)}^{2}+K_{1}\right)/2$ holds.

### 4.3 Results on the first phase of the framework

For each chemical property π , we construct a prediction function by one of the following four methods (i)–(iv) and compare their results.

(i) LLR: use lasso linear regression on the set $D_{\pi }^{\left(1\right)}$ of linear descriptors (see Zhu et al. (2022b) for the details of the implementation);
(ii) ANN: use ANN on the set $D_{\pi }^{\left(1\right)}$ of linear descriptors (see Zhu et al. (2022b) for the details of the implementation);
(iii) ALR: use adjustive linear regression on the set $D_{\pi }^{\left(1\right)}$ of linear descriptors (see Zhu et al. (2022c) for the details of the implementation); and
(iv) R-MLR: apply our method (see Zhu et al. (2022a)) of reducing descriptors to the set $D_{\pi }^{\left(1\right)}\cup D_{\pi }^{\left(2\right)}$ of linear and quadratic descriptors, and use multi-linear regression for the resulting set of descriptors.

For methods (i)–(iii), we use the same implementation described in Zhu et al. (2022b) and Zhu et al. (2022c) and omit the details.

In method (iv), we apply LLR-Reduce $\left(C_{\pi },D_{\pi }^{\left(1\right)}\cup D_{\pi }^{\left(2\right)}\right)$ to compute $\tilde{D}\subset D_{\pi }^{\left(1\right)}\cup D_{\pi }^{\left(2\right)}$ of size 5000 if $|D_{\pi }^{\left(1\right)}\cup D_{\pi }^{\left(2\right)}|>5000$ for a monomer property π . In the other case, we set $\tilde{D}≔D_{\pi }^{\left(1\right)}\cup D_{\pi }^{\left(2\right)}$ . Then, apply Select-Des-set $\left(C_{\pi },\tilde{D}\right)$ to get $D^{*}\subseteq \tilde{D}$ , which is used to construct prediction functions based on MLR.

Results of the learning experiments are listed in Tables 3, 4, where:

- π : the property name;
- Λ : the chemical element set of $C_{\pi }$ ;
- LLR, ANN, ALR, R-MLR: the median of test $R^{2}$ score in ten times 5-fold cross-validations for functions obtained by methods (i), (ii), (iii), and (iv), respectively;
- the best $R^{2}$ score for each property among LLR, ANN, ALR, and R-MLR is indicated by “*”;
- $K_{1}^{*},K_{2}^{*}$ : the number $K_{1}^{*}$ of reduced linear descriptors and the number $K_{2}^{*}$ of reduced quadratic descriptors in $D^{*}$ used in MLR.

**TABLE 3:** Results of constructing prediction functions for monomers.

π	Λ	LLR	ANN	ALR	R-MLR	$K_{1}^{*},K_{2}^{*}$
BHL	$\Lambda_{7}$	0.483	0.622	0.265	*0.659	0, 27
BP	$\Lambda_{2}$	0.599	0.765	0.816	*0.935	1, 59
BP	$\Lambda_{7}$	0.663	0.720	0.832	*0.899	0, 38
CP	$\Lambda_{5}$	0.555	0.727	0.690	*0.841	0, 67
CT	$\Lambda_{2}$	0.037	0.357	0.900	*0.937	1, 47
CT	$\Lambda_{5}$	0.048	0.357	*0.895	0.860	0, 13
DC	$\Lambda_{2}$	0.489	0.651	0.488	*0.908	0, 58
DC	$\Lambda_{7}$	0.574	0.622	0.602	*0.829	0, 26
ET	$\Lambda_{7}$	0.132	0.479	0.464	*0.996	0, 13
FP	$\Lambda_{2}$	0.589	0.746	0.719	*0.899	0, 42
FP	$\Lambda_{7}$	0.571	0.745	0.684	*0.846	0, 32
FIMLO	$\Lambda_{16}$	0.819	0.928	0.604	*0.949	0, 77
HV	$\Lambda_{2}$	0.864	0.778	0.816	*0.970	0, 22
KOV	$\Lambda_{1}$	0.677	0.727	0.838	*0.953	2, 19
KOW	$\Lambda_{2}$	0.953	0.952	0.964	*0.967	0, 55
KOW	$\Lambda_{8}$	0.927	0.937	*0.952	0.950	0, 64
LP	$\Lambda_{2}$	0.856	0.867	0.844	*0.928	0, 89
LP	$\Lambda_{8}$	0.840	0.859	0.807	*0.914	0, 109
MP	$\Lambda_{2}$	0.810	0.800	0.831	*0.873	0, 51
MP	$\Lambda_{8}$	0.810	0.820	0.807	*0.898	0, 58
OPTR	$\Lambda_{2}$	0.825	0.918	0.876	*0.970	0, 85
OPTR	$\Lambda_{4}$	0.825	0.878	0.870	*0.970	0, 69
RFIDT	$\Lambda_{10}$	0.000	0.453	0.425	*0.775	0, 43
SL	$\Lambda_{2}$	0.808	0.848	0.784	*0.894	0, 82
SL	$\Lambda_{8}$	0.808	0.861	0.828	*0.897	0, 74
SFT	$\Lambda_{3}$	0.927	0.859	0.847	*0.941	0, 36
VIS	$\Lambda_{3}$	0.893	0.929	0.911	*0.973	0, 43
HOMO	$\Lambda_{9}$	*0.841	0.689	0.689	0.804	0, 87
LUMO	$\Lambda_{9}$	0.841	0.860	0.833	*0.920	0, 102
GAP	$\Lambda_{9}$	0.784	0.795	0.755	*0.876	0, 83
ALPHA	$\Lambda_{9}$	0.961	0.888	0.953	*0.980	0, 104
CV	$\Lambda_{9}$	0.970	0.911	0.966	*0.978	0, 83
MU	$\Lambda_{9}$	0.367	0.409	0.403	*0.645	0, 112

**TABLE 4:** Results of constructing prediction functions for polymers. The negative value shows that the respective model is arbitrarily worse.

π	Λ	LLR	ANN	ALR	R-MLR	$K_{1}^{*},K_{2}^{*}$
AMD	$\Lambda_{2}$	0.914	0.885	*0.933	0.906	0, 5
AMD	$\Lambda_{13}$	0.918	0.824	0.917	*0.953	0, 6
CHAR	$\Lambda_{2}$	0.210	0.642	0.863	*0.938	0, 10
CHAR	$\Lambda_{12}$	0.088	0.640	0.835	*0.924	0, 9
CHAR	$\Lambda_{6}$	−0.073	0.527	0.766	*0.950	0, 12
DEIC	$\Lambda_{12}$	0.761	0.641	0.918	*0.956	3, 41
DISF	$\Lambda_{13}$	0.623	0.801	0.308	*0.906	1, 23
PRM	$\Lambda_{2}$	0.801	0.801	0.505	*0.967	0, 26
PRM	$\Lambda_{13}$	0.784	0.735	0.489	*0.977	0, 34
RFIDP	$\Lambda_{11}$	0.104	0.423	0.853	*0.962	2, 52
RFIDP	$\Lambda_{14}$	0.373	0.560	0.848	*0.953	2, 43
RFIDP	$\Lambda_{15}$	0.346	0.492	0.883	*0.947	5, 53
TG	$\Lambda_{2}$	0.902	0.883	0.923	*0.958	1, 33
TG	$\Lambda_{7}$	0.894	0.860	0.927	*0.957	0, 32

The computation times of finding $D^{*}$ and constructing functions by our method (iv) were in the ranges of $\left[80,4\times 10^{4}\right]$ seconds and [0.03,0.46] seconds, respectively.

Tables 3, 4 show that method (iv) significantly increased the learning performance of several properties and achieved the best scores among methods (i)–(iii) for 43 of 47 data sets. We also noticed that most of the selected descriptors in $D^{*}$ are quadratics, which confirms the effectiveness of the proposed quadratic descriptors.

### 4.4 Results on the second phase of the framework

To execute the second phase, we used a set of seven instances $I_{\text{a}}$ , $I_{\text{b}}^{i},i\in \left[1,4\right]$ , $I_{\text{c}}$ , and $I_{\text{d}}$ based on the seed graphs prepared by Zhu et al. (2022b). We here present their seed graphs $G_{C}$ (see Zhu et al. (2022a)) for the details of $I_{\text{a}}$ , $I_{\text{b}}^{i},i\in \left[1,4\right]$ , $I_{\text{c}}$ , and $I_{\text{d}}$ ).

**FIGURE 2:**
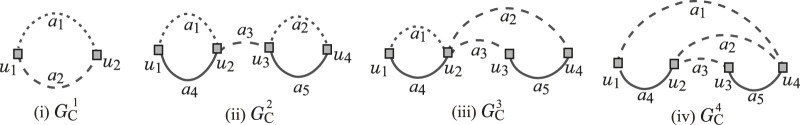
(i) Seed graph $G_{C}^{1}$ for $I_{\text{b}}^{1}$ and $I_{\text{d}}$ ; (ii) seed graph $G_{C}^{2}$ for $I_{\text{b}}^{2}$ ; (iii) seed graph $G_{C}^{3}$ for $I_{\text{b}}^{3}$ ; (iv) seed graph $G_{C}^{4}$ for $I_{\text{b}}^{4}$ .

The seed graph $G_{C}^{1}$ of $I_{\text{b}}^{1}$ (resp., $G_{C}^{i},\;i=2,3,4$ of $I_{\text{b}}^{i},\;i=2,3,4$ ) is illustrated in Figure 2.

Instance $I_{\text{c}}$ is introduced to infer an intermediate graph $C^{†}$ , which preserves

**FIGURE 3:**
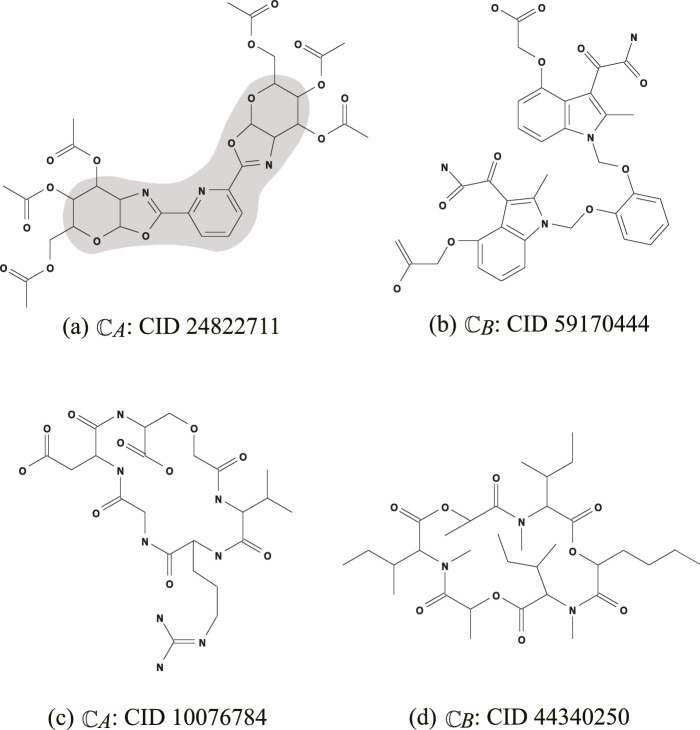
An illustration of chemical compounds for instances $I_{\text{c}}$ and $I_{\text{d}}$ : **(A)** $C_{A}$ : CID 24822711; **(B)** $C_{B}$ : CID 59170444; **(C)** $C_{A}$ : CID 10076784; **(D)** $C_{B}$ : CID 44340250, where hydrogens are omitted.

- the core part of $C_{A}$ : CID 24822711 given in Figure 3A; and

- the frequencies of all edge-configurations of $C_{B}$ : CID 59170444 given in Figure 3B.The seed graph $G_{C}$ of this instance is depicted in gray in Figure 3A).

Instance $I_{\text{d}}$ has been introduced in order to infer a chemical graph $C^{†}$ such that

- $C^{†}$ is monocyclic (where the seed graph of $I_{\text{d}}$ is given by $G_{C}^{1}$ in Figure 2(i)); and

- the frequency vector of edge configurations in $C^{†}$ is a vector obtained by merging those of chemical graphs $C_{A}$ : CID 10076784 and $C_{B}$ : CID 44340250 in Figures 3C, D, respectively.

#### 4.4.1 Solving an MILP for the inverse problem

We executed the stage of solving an MILP to infer a chemical graph for two properties π∈ {Bp, Dc}.

For the MILP formulation $M_{f,\eta ,\sigma }$ , we use the prediction function η for each π∈ {Bp, Dc} by method (iv), R-MLR that attained the median test $R^{2}$ in Table 3. To solve an MILP with the formulation, we used CPLEX version 12.10. Tables 5, 6 show the computational results of the experiment in this stage for the two properties, where we denote the following:

- $n_{LB}$ : a lower bound on the number of non-hydrogen atoms in a chemical graph C to be inferred;
- ${\underline{y}}^{*},\;{\bar{y}}^{*}$ : lower and upper bounds ${\underline{y}}^{*},{\bar{y}}^{*}\in R$ on the value a(C) of a chemical graph C to be inferred;
- # v (resp., # c): the number of variables (resp., constraints) in the MILP;
- I-time: the time (sec.) to solve the MILP;
- n : the number $n\left(C^{†}\right)$ of non-hydrogen atoms in the chemical graph $C^{†}$ inferred by solving the MILP;
- $n^{int}$ : the number $n^{int}\left(C^{†}\right)$ of interior vertices in the chemical graph $C^{†}$ ; and
- η : the predicted property value $\eta \left(f\left(C^{†}\right)\right)$ of the chemical graph $C^{†}$ .

**TABLE 5:** Results of inferring a chemical graph $C^{†}$ and generating recombination solutions for Bp with $\Lambda_{7}$ .

inst.	$n_{LB}$	${\underline{y}}^{*},\;{\bar{y}}^{*}$	# v	# c	I-time	n	$n^{int}$	η	D-time	C -LB	#C
$I_{\text{a}}$	30	225, 235	10,502	10,240	4.29	49	26	233.92	0.072	3	3
$I_{\text{b}}^{1}$	35	285, 295	10,507	7,793	2.27	35	10	286.52	0.034	6	6
$I_{\text{b}}^{2}$	45	365, 375	13,000	10,913	11.9	49	25	370.70	0.14	3202	100
$I_{\text{b}}^{3}$	45	305, 315	12,788	10,920	7.07	48	25	309.39	0.22	6,304	100
$I_{\text{b}}^{4}$	45	260, 270	12,576	10,928	10.7	49	27	266.26	0.17	376	100
$I_{\text{c}}$	50	340, 350	7,515	8,270	0.867	50	33	344.98	0.019	2	2
$I_{\text{d}}$	40	320, 330	6,135	7,773	8.22	45	23	329.85	8.3	6,733,440	100

**TABLE 6:** Results of inferring a chemical graph $C^{†}$ and generating recombination solutions for Dc with $\Lambda_{7}$ .

inst.	$n_{LB}$	${\underline{y}}^{*},\;{\bar{y}}^{*}$	# v	# c	I-time	n	$n^{int}$	η	D-time	C -LB	#C
$I_{\text{a}}$	30	0.55, 0.60	10,194	9787	3.91	41	25	0.558	0.069	2	2
$I_{\text{b}}^{1}$	35	1.10, 1.15	10,415	7,368	4.73	35	11	1.104	0.10	16	16
$I_{\text{b}}^{2}$	45	6.00, 6.05	12,976	10,481	57.4	45	25	6.04	0.12	2040	100
$I_{\text{b}}^{3}$	45	1.45, 1.50	2,767	10,488	39.7	49	26	1.488	0.28	21,600	100
$I_{\text{b}}^{4}$	45	6.10, 6.15	12,558	10,494	26.4	46	25	6.10	0.027	2	2
$I_{\text{c}}$	50	12.35, 12.40	7,207	7,819	1.75	50	34	12.38	0.020	2	2
$I_{\text{d}}$	40	3.15, 3.20	5827	7,325	14.9	41	23	3.199	0.079	18,952	100

**FIGURE 4:**
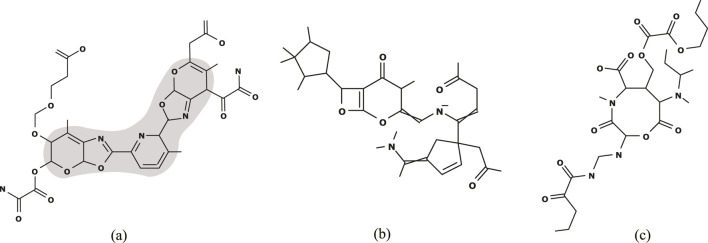
**(A)** $C^{†}$ with $\eta \left(f\left(C^{†}\right)\right)=344.98$ inferred from $I_{\text{c}}$ with $\left({\underline{y}}^{*},{\bar{y}}^{*}\right)=\left(340,350\right)$ of Bp; **(B)** $C^{†}$ with $\eta \left(f\left(C^{†}\right)\right)=0.558$ inferred from $I_{\text{a}}$ with $\left({\underline{y}}^{*},{\bar{y}}^{*}\right)=\left(0.55,0.60\right)$ of Dc; and **(C)** $C^{†}$ with $\eta \left(f\left(C^{†}\right)\right)=3.199$ inferred from $I_{\text{d}}$ with $\left({\underline{y}}^{*},{\bar{y}}^{*}\right)=\left(3.15,3.20\right)$ of Dc.

Figure 4A illustrates the chemical graph $C^{†}$ inferred from $I_{\text{c}}$ with $\left({\underline{y}}^{*},{\bar{y}}^{*}\right)=\left(340,350\right)$ of Bp in Table 5.

Figure 4B (resp., Figure 4C) illustrates the chemical graph $C^{†}$ inferred from $I_{\text{a}}$ with $\left({\underline{y}}^{*},{\bar{y}}^{*}\right)=\left(0.55,0.60\right)$ (resp., $I_{\text{d}}$ with $\left({\underline{y}}^{*},{\bar{y}}^{*}\right)=\left(3.15,3.20\right)$ ) of Dc in Table 6.

In this experiment, we prepared several different types of instances: instances $I_{\text{a}}$ and $I_{\text{c}}$ have restricted seed graphs, the other instances have abstract seed graphs, and instances $I_{\text{c}}$ and $I_{\text{d}}$ have restricted sets of fringe trees. From Tables 5, 6, we observe that an instance with many variables and constraints takes more running time than those with a smaller size in general. All instances in this experiment are solved in a few seconds to approximately 60 s with our MILP formulation.

#### 4.4.2 Generating recombination solutions

Let $C^{†}$ be a chemical graph obtained by solving the MILP $M_{f,\eta ,\sigma }$ for the inverse problem. We here execute a stage of generating recombination solutions $C^{*}\in G_{\sigma }$ of $C^{†}$ such that $f\left(C^{*}\right)=x^{*}=f\left(C^{†}\right)$ .

We execute an algorithm for generating chemical isomers of $C^{†}$ up to 100 when the number of all chemical isomers exceeds 100. For this, we use a dynamic programming algorithm (Zhu et al., 2022b). The algorithm first decomposes $C^{†}$ into a set of acyclic chemical graphs. Next, it replaces each acyclic chemical graph T with another acyclic chemical graph $T^{\prime }$ that admits the same feature vector as that of T , and finally, it assembles the resulting acyclic chemical graphs into a chemical isomer $C^{*}$ of $C^{†}$ . The algorithm can compute a lower bound on the total number of all chemical isomers $C^{†}$ without generating all of them.

Tables 5, 6 show the computational results of the experiment in this stage for the two properties π∈ {Bp, Dc}, where we denote the following:

- D-time: the running time (s) to execute the dynamic programming algorithm to compute a lower bound on the number of all chemical isomers $C^{*}$ of $C^{†}$ and generate all (or up to 100) chemical isomers $C^{*}$ ;
- C -LB: a lower bound on the number of all chemical isomers $C^{*}$ of $C^{†}$ ; and
- #C : the number of all (or up to 100) chemical isomers $C^{*}$ of $C^{†}$ generated in this stage.

From Tables 5, 6, we observe the running time and the number of generated recombination solutions in this stage.

The chemical graph $C^{†}$ in $I_{\text{b}}^{2}$ , $I_{\text{b}}^{3}$ , and $I_{\text{d}}$ admits a large number of chemical isomers $C^{*}$ in some cases, where a lower bound C -LB on the number of chemical isomers is derived without generating all of them. For the other instances, the running time for generating up to 100 target chemical graphs in this stage is less than 0.03 s. For some chemical graphs $C^{†}$ , the number of chemical isomers found by our algorithm was small. This is because some of the acyclic chemical graphs in the decomposition of $C^{†}$ have no alternative acyclic chemical graph other than the original one.

#### 4.4.3 Generating neighbor solutions

Let $C^{†}$ be a chemical graph obtained by solving the MILP $M_{f,\eta ,\sigma }$ for the inverse problem. We executed a stage of generating neighbor solutions of $C^{†}$ .

We select an MILP for the inverse problem with a prediction function η such that a solution $C^{†}$ of the MILP admits only two isomers $C^{*}$ in the stage of generating recombination solutions; that is, instance $I_{\text{c}}$ for property Bp with $\Lambda_{7}$ and instances $I_{\text{a}}$ , $I_{\text{b}}^{4}$ and $I_{\text{c}}$ for property Dc with $\Lambda_{7}$ .

In this experiment, we add to the MILP $M_{f,\eta ,\sigma }$ an additional set Θ of two linear constraints on linear and quadratic descriptors as follows. For the two constraints, we use the prediction functions $\eta_{\pi }$ constructed by R-MLR for properties π∈ { Lp, Sl} with $\Lambda_{8}$ in Table 3.

Let $D_{\pi }^{*}$ denote the set of descriptors selected in the construction of prediction function for properties π∈ { Bp, Dc} with $\Lambda_{7}$ and π∈ { Lp, Sl} with $\Lambda_{8}$ in Table 3, and let $D_{\pi }^{\text{union}},\pi \in$ { Bp, Dc} denote the union $D_{\pi }^{*}\cup D_{\text{LP}}^{*}\cup D_{\text{SL}}^{*}$ .

We regard each of η _Lp_ and η _Sl_ as a function from $R^{\left|D_{\pi }^{\text{union}}\right|}$ to R for π∈ { Bp, Dc}. We set $p_{\text{dim}}≔2$ and let Θ consist of two linear constraints $\theta_{1}≔\eta$ _Lp_ and $\theta_{2}≔\eta$ _Sl_. We set δ≔0.1 or 0.05, which defines a two-dimensional grid space where $C^{†}$ is mapped to the origin (see Azam et al. (2021a) for the details on the neighbors). In these experiments, we check the feasibility of 48 neighbors of the origin $C^{†}$ in a grid in an increasing order w.r.t. the distance. The time limit of the solver is set to be 300 s. We do not check the feasibility of a neighbor z and ignore it if there exists a neighbor $z^{\prime }$ for which the MILP formulation is infeasible and $z^{\prime }$ is closer to $C^{†}$ than z .The results of these experiments are listed in Table 7 where

- (inst., π ): specification I and property π ;
- n : the number of atoms in the hydrogen-suppressed test instance;
- δ : the size of a sub-region in the grid;
- #sol: the number of new graphs inferred from 48 neighbors;
- #infs: the number of infeasible neighbors;
- #ign: the number of ignored neighbors;
- #TO: the number of neighbors for which the running time of the solver exceeds the time limit.

**TABLE 7:** Results of generating neighbor solutions of $C^{†}$ .

(inst., π )	n	δ	#sol	#infs	#ign	#TO
( $I_{\text{c}}$ , Bp)	50	0.1	5	1	3	39
( $I_{\text{a}}$ , Dc)	30	0.1	40	1	0	7
( $I_{\text{b}}^{4}$ , Dc)	45	0.1	2	0	0	46
( $I_{\text{c}}$ , Dc)	40	0.05	0	0	0	48

The branch-and-bound method for solving an MILP sometimes takes an extremely large execution time for the same size of instances. We introduce a time limit to bound an entire running time to skip such instances when testing the feasibility of neighbors in $N_{0}$ . From Table 7, we observe that some number of neighbor solutions of the solution $C^{†}$ to the MILP $M_{f,\eta ,\sigma }$ could be generated for each of the four instances.

## 5 Conclusion

In the framework of inferring chemical graphs, the descriptors of a prediction function were mainly defined to be the frequencies of local graph structures in the two-layered model, and such a definition was important to derive a compact MILP formulation for inferring a desired chemical graph. To improve the performance of prediction functions in the framework, this article introduced a multiplication of two of these descriptors as a new descriptor and examined the effectiveness of the new set of descriptors. For this, we designed a method for reducing the size of a descriptor set to not lose the learning performance in constructing prediction functions and gave a compact formulation to compute a product of two values in an MILP. From the results of our computational experiments, we observe that a prediction function constructed by our new method performs considerably better than the prediction functions constructed by the previous methods for several chemical properties. Furthermore, the modified MILP, with the computation of quadratic descriptors, was able to infer desired chemical graphs with up to 50 non-hydrogen atoms.

## Data Availability

The original contributions presented in the study are included in the article/Supplementary Material; further inquiries can be directed to the corresponding author.

## Glossary

Alpha: isotropic polarizability
ALR: adjustive linear regression
AmD: experimental amorphous density
ANN: artificial neural network
BHL: biological half life
Bp: boiling point
ChaR: characteristic ratio
Cp: critical pressure
Ct: critical temperature
Cv: heat capacity at 298.15 K
Dc: dissociation constants
DieC: dielectric constant
DisF: dissipation factor
EDPA: electron density on the most positive atom
ET: entropy
FlmLO: flammable limits lower of organics
Fp: flash point in closed cup
Gap: the energy difference between HOMO and LUMO
Ha: heat of atomization
Hc: heat of combustion
HcL: heat capacity in liquid
HcS: heat capacity in solid
Hf: heat of formation
HOMO: energy of highest-occupied molecular orbital
Hv: heat of vaporization
Kov: Kovats retention index
Kow: octanol/water partition coefficient
LLR: lasso linear regression
Lp: lipophilicity
LUMO: energy of lowest-unoccupied molecular orbital
MILP: mixed-integer linear programming
MLR: multidimensional linear regression
MlV: mol volume
Mp: melting point
Mr: molar refraction at 20° degree
mu: electric dipole moment
OptR: optical rotation
Prm: permittivity
QSAR: quantitative structure–activity relationship
RfIdP: refractive index of polymers
RfIdT: refractive index of trees
SfT: surface tension
Sl: solubility
Tg: glass transition
U0: internal energy at 0 K
Vd: vapor density
Vis: viscosity
Vp: vapor pressure
